# Atomically resolved three-dimensional structures of electrolyte aqueous solutions near a solid surface

**DOI:** 10.1038/ncomms12164

**Published:** 2016-07-15

**Authors:** Daniel Martin-Jimenez, Enrique Chacon, Pedro Tarazona, Ricardo Garcia

**Affiliations:** 1 Instituto de Ciencia de Materiales de Madrid, CSIC, c/ Sor Juana Ines de la Cruz 3, 28049 Madrid, Spain; 2 Department Física Teórica de la Materia Condensada, IFIMAC Condensed Matter Physics Center, UAM, 28049 Madrid, Spain

## Abstract

Interfacial liquid layers play a central role in a variety of phenomena ranging from friction to molecular recognition. Liquids near a solid surface form an interfacial layer where the molecular structure is different from that of the bulk. Here we report atomic resolution three-dimensional images of electrolyte solutions near a mica surface that demonstrate the existence of three types of interfacial structures. At low concentrations (0.01–1 M), cations are adsorbed onto the mica. The cation layer is topped by a few hydration layers. At higher concentrations, the interfacial layer extends several nanometres into the liquid. It involves the alternation of cation and anion planes. Fluid Density Functional calculations show that water molecules are a critical factor for stabilizing the structure of the interfacial layer. The interfacial layer stabilizes a crystal-like structure compatible with liquid-like ion and solvent mobilities. At saturation, some ions precipitate and small crystals are formed on the mica.

## Abstract (web summary)

Liquids near a solid surface form an interfacial layer where the molecular structure is different from the bulk. Here the authors report atomic resolution three-dimensional images of electrolyte solutions that demonstrate the existence of three types of interfacial structures as a function of concentration.

## Graphical abstract

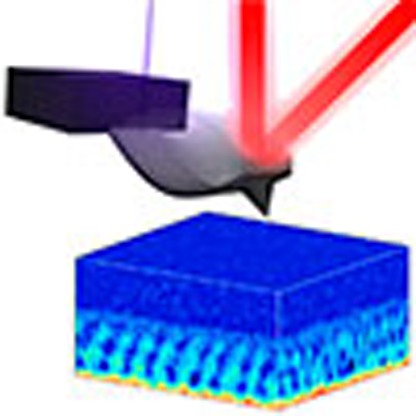

Interfacial liquid layers are involved in a wide range of phenomena such as wetting^1^,^2^, adhesion^3^, surface electrochemistry^4^, nanorheology^5^, nanolithography^6^, biomineralization^7^, protein dynamics^8^ or protein folding and molecular recognition^9^. It is known that liquids near a solid surface form an interfacial layer where the molecular structure is different from the organization of molecules in the bulk^10^,^11^. The structure and the associated dynamics of the layer are controlled by the discrete character of the ions and solvent molecules. The number density of liquid molecules next to a solid surface might oscillate with respect to the bulk density and with a periodicity close to one molecular diameter. Those oscillations have been studied with computer simulations^12^ and Classical Fluid Density Functional Theory (CF-DFT)^13^,^14^. The oscillations could extend several molecular diameters into the liquid and give rise to a periodic force when two surfaces confining the liquid are brought together into a few molecular diameter thick gaps^15^,^16^,^17^.

More than 30 years have passed since the first observation of periodic hydration forces between mica surfaces in dilute electrolyte solutions^15^. A variety of experimental and theoretical methods have been applied to study interfacial liquids, including X-ray reflectivity^18^, X-ray absorption spectroscopy^19^, various force spectroscopies^17^,^20^,^21^, first-principle^22^ and molecular dynamics calculations^23^,^24^. In particular, electronic DFT simulations have shown that a three-dimensional (3D) atomic structure should appear at the liquid adjacent to a solid crystalline surface^25^; however, to this date its direct experimental observation has remained elusive. Recent developments in high-resolution atomic force microscopy (AFM) have imaged the existence of a few hydration layers on top of a mica surface^26^,^27^, ionic crystals^28^,^29^, lipid headgroups^30^ and protein surfaces^31^. AFM images have also revealed with atomic resolution the adsorption from a solution of Na^+^, Rb^+^, Mg^2+^ or Ca^2+^ ions on different surfaces^32^,^33^,^34^,^35^. Those results indicate that advanced force microscopy methods could be applied to determine the 3D atomic structure of liquids near solid surfaces.

Muscovite mica is a suitable substrate to study the interfacial structure of alkali halide aqueous (aq.) solutions because it is atomically flat and it has a net negative surface charge on cleavage. The electrostatic interaction of the alkali cations with mica would favour their attachment. The basal plane (cleaved) of the mica (001) has a hexagonal structure similar to the (111) planes of NaCl and KCl crystals. The mica lattice constant in the (001) plane (0.52 nm) is higher than the corresponding for NaCl(111) and KCl(111), 0.398 and 0.444 nm, respectively.

Here we provide atomically resolved 3D images of solid-electrolyte interfaces with several nanometres in depth. The images acquired at 300 K allow us to characterize the interfacial structures as a function of the electrolyte concentration near a mica surface. At low-to-moderate molarities (<1 M), cations are adsorbed on mica^33^. The monolayer of cations is topped by a few hydration layers. At high molarities, the interfacial layer shows an epitaxial-like growth structure that extends several nanometres into the liquid. The ordered layer involves an alternation of cation and anion planes. A significant number of water molecules are placed between the planes to balance the electrostatic interactions and stabilize the structure. We show that a stable crystalline-like structure is compatible with liquid-like ion and solvent mobilities. At saturation, some ions precipitate and form nano and micro crystals on the mica.

## Results

### 3D AFM

**Figure 1:**
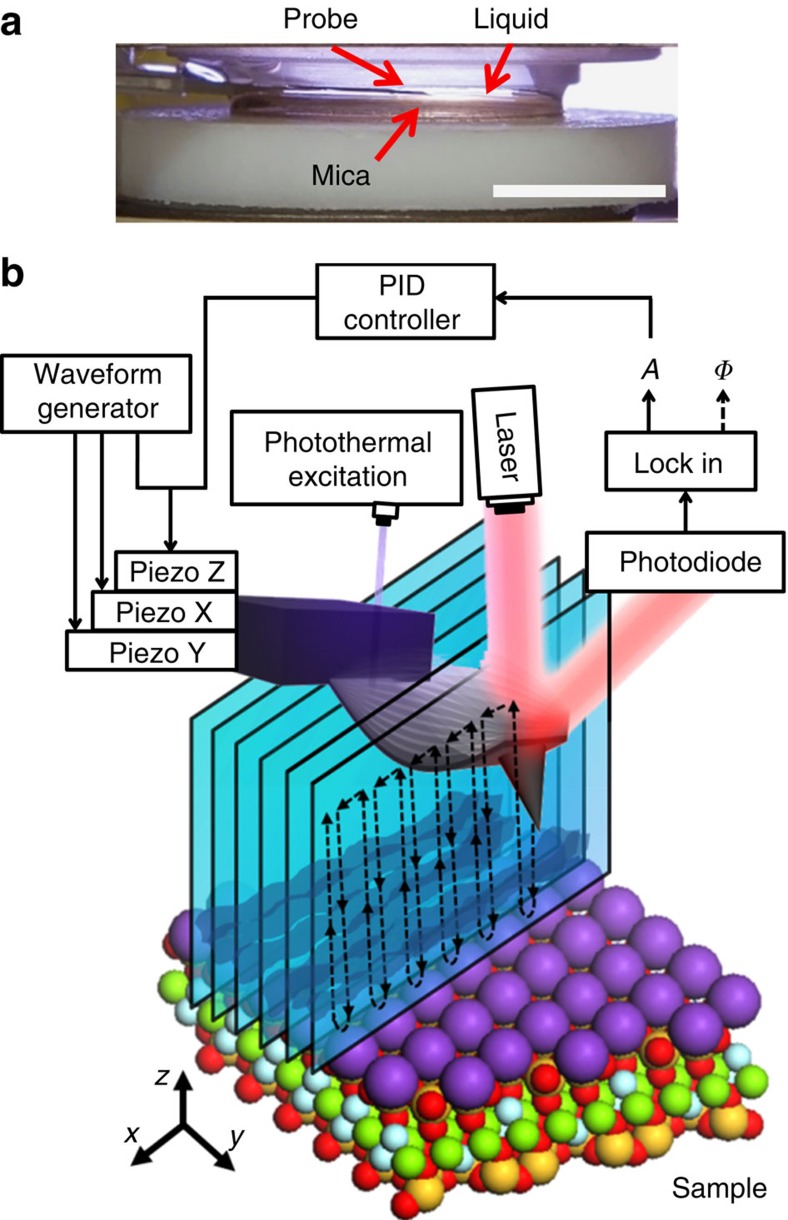
Fluid cell and 3D AFM scheme. (**a**) AFM fluid cell. A thin mica substrate is glued onto a Teflon plate (white disk) while a transparent glass surface closes the top of the liquid cell. The thickness of the electrolyte solution is about 1–2 mm. The AFM cantilever is inside the liquid. (**b**) Scheme of the tip displacements, cantilever excitation and detection and observables used to acquire a 3D image of a solid-liquid interface. Scale bar, 5 mm (**a**).

Figure 1a shows a photograph of the fluid cell. An electrolyte aqueous solution is confined between a mica surface and a glass surface (top of the panel). The AFM cantilever is immersed in the liquid (not seen). The tip explores the 3D solid–liquid interface by acquiring a sequential series of *xz* planes. In each plane, the tip follows the trajectory depicted in Fig. 1b. The AFM was operated with an amplitude modulation AFM^36^ feedback control (see Methods section). Amplitude modulation AFM offers a robust and sensitive feedback mechanism against changes in the tip environment. The implementation of 3D AFM with an amplitude modulation feedback has been crucial to expand the depth of the 3D AFM images to the 10 nm range. This is in contrast with previous 3D AFM images^26^,^30^. Those images were obtained with a frequency modulation feedback where the 3D range was in the sub-2 nm range^26^,^30^. A 3D AFM image is obtained by plotting the changes of the tip observables, either the amplitude (*A*) or the phase shift (*ϕ*), as a function of the spatial coordinates (*x*, *y*, *z*).

**Figure 2:**
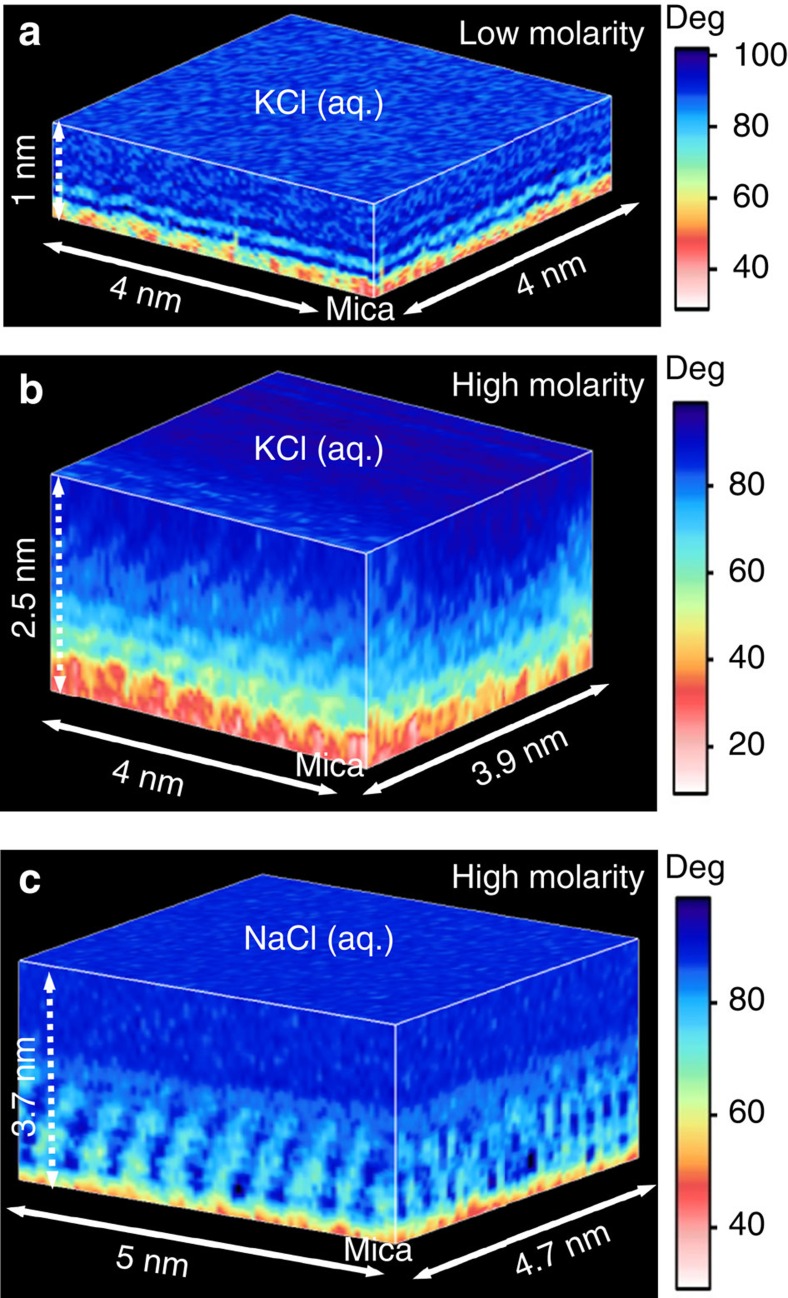
Three-dimensional images of mica-electrolyte solution interfaces. (**a**) 3D AFM image of a KCl (aq.) solution (0.2 M KCl). The image shows a monolayer of adsorbed K^+^ ions (light red) topped by two hydration layers (lighter stripes). The hydration layers (∼0.3 nm thick) follow the atomic corrugation of the surface. (**b**) 3D AFM image of a mica-KCl (aq.) interface (∼4 M). The interface is divided in two main regions, an ordered liquid layer extending up to 2 nm from the mica and the bulk solution above it. (**c**) 3D AFM image of a mica-NaCl (aq.) interface. Atomic scale order is seen in *xy, xz* and *yz* planes. These 3D maps show the variations of the phase shift of the tip's oscillation. Supplementary Movies 1 (52 s), 2 (105 s) and 3 (105 s) include the complete 3D data, respectively, of **a**–**c**. The 3D AFM experiments were performed at ∼300 K.

Several 3D AFM images of the mica-electrolyte solution interfaces, two corresponding to different mica-KCl (aq.) interfaces and the third to a mica-NaCl (aq.) interface are shown in Fig. 2. The whole sequence of images is showed in the Supplementary Information (Supplementary Movies 1, 2 and 3). Figure 2a shows an image of a mica-KCl aqueous interface (0.2 M KCl). The 3D AFM shows an atomic scale corrugation on the mica (light red region) and an alternation of ultrathin layers (∼0.3 nm) following the atomic corrugation. Calculations (below) assign the atomic corrugation to a monolayer of cations adsorbed on the mica while the ultrathin layers represent variations of the water density. Figure 2b shows an image of a mica-KCl (aq.) interface (∼4 M KCl). The image shows an ordered interface that extends several nanometres into the liquid. The ordered structure resembles an epitaxial growth process because the periodicity in the *xy* plane (≈0.5 nm) follows the periodicity of the mica substrate (0.52 nm).

The data discussed here is focused on mica-KCl (aq.) interfaces; however, we have included images from a mica-NaCl (aq.) (Fig. 2c) and mica-RbCl interfaces (Supplementary Fig. 1) to illustrate that the observed phenomenology is not restricted to KCl solutions. It also applies to other alkali halide electrolyte solutions. The Supplementary Movies 1, 2, 3 are associated with the 3D AFM images from Fig. 2. The movies have been acquired in 52, 105 and 105 s, respectively. Those values indicate that the observed ordered liquid layers are stable over several minutes.

### Interfacial organization of electrolyte aqueous solutions

**Figure 3:**
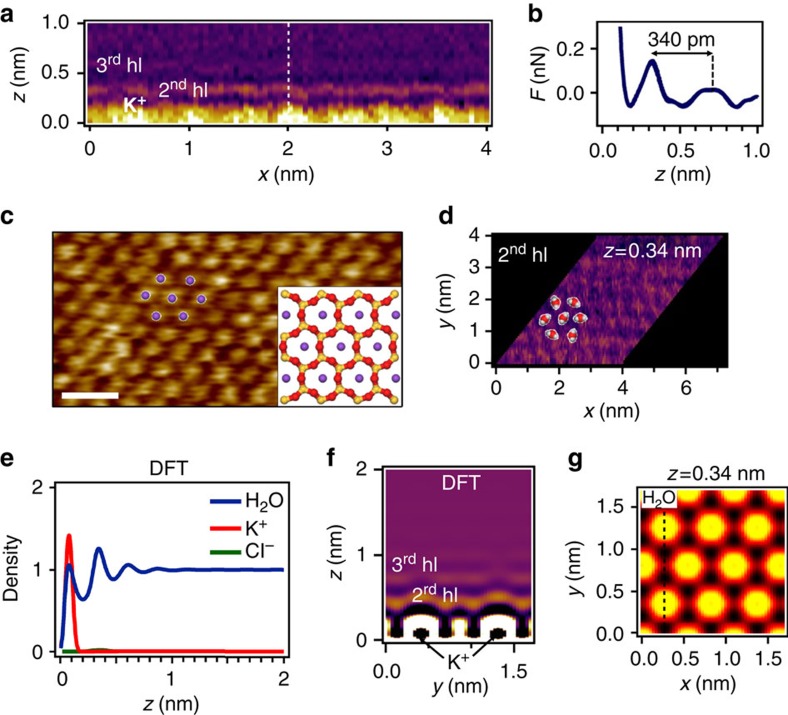
Atomic scale structure of mica-KCl interfaces at low molarities. (**a**) *xz* frame (raw data). At low to moderate salt concentrations (0.01–1 M), the ordered layer is very thin (below 1.0 nm). It is formed by a single cation monolayer on top of the mica lattice and a few hydration layers. (**b**) The force curve across the dashed line of **a** shows two hydration layers about 0.34 nm apart. (**c**) *xy* frame taken at *z*=0 nm. In the experimental images, the *z*=0 nm is chosen to be located at the interstitial position between two K^+^. This assumption facilitates the direct comparison between theory and CF-DFT data. The image shows the hexagonal structure of the K^+^ on mica. The inset shows the structure of mica (001); Oxygen (red), silicon or aluminium (yellow) and the adsorbed K ions (purple). Si and Al are in a 3 to 1 ratio. The plane of the K atoms lies above the O and Si planes. (**d**) *xy* frame acquired at *z*=0.34 nm. The image shows the structure of the water molecules in the 2^nd^ hydration layer. The origin of *z* is chosen at the mica surface (minima in **a**). (**e**) CF-DFT results of the number density perpendicular to the mica surface (*z* direction). At each *z* point, the density profile represents the average value over the corresponding *xy* plane. The density profiles are normalized with respect to the bulk number density of water. (**f**) CF-DFT *xz* total number density map of the mica-KCl (aq.) interface. This map is calculated for the *x* position marked with a dashed line in **g**. (**g**) CF-DFT *xy* map at *z*=0.34 nm. At this position the map mainly reflects the arrangement of the water molecules. Scale bar, 1 nm (**c**).

The structure, extension and periodicities of the ordered liquid layer depend on the salt concentration. At low to moderate concentrations (0.01–1 M), the 3D AFM data for a KCl (aq.) solution (Fig. 3a–c) show the presence of adsorbed K^+^ cations on the mica surface and a few hydration layers (∼0.3 nm thick) above them (Fig. 3a). Figure 3b shows that the force sensed by the tip along the dashed line shown in Fig. 3a alternates repulsive and attractive regions. The curve shows a repulsive region when the tip is on top of an adsorbed cation and two local repulsive maxima ∼0.34 nm apart. To determine the force curve from the 3D AFM data is complex and tedious because a single force value *F*(*x*_i_,*y*_j_,*z*_k_) requires to know the dependence of the observables with *z* from *z*_k_ to ∞. We have adapted the force inversion methods deduced for 1D AFM measurements^37^,^38^. The main steps to deduce the force curve from the 3D AFM observables are illustrated in Supplementary Fig. 2. The measured force depends on the hydration layer order. We report forces between 50–150 pN (Fig. 3b). Those values are comparable to the data provided by molecular dynamics simulations and experiments on a calcite–water interface^39^.

Our CF-DFT calculation reveals the main factors in the above experiments. We do not aim to provide a full quantitative description of the experimental data, nor to give the most realistic description of the ionic solutions, but rather to reproduce and explain the phenomena semi-quantitatively. The theoretical difficulties associated with a realistic description of the ionic interactions are not crucial to predict the qualitative behaviour of the system. The most relevant factor is to observe that by increasing the salt concentration we should get closer to its crystallization phase transition, which may be represented by short-ranged effective interactions. The interaction with the mica is modelled to have a nearly complete monolayer of cations at low salt concentrations (0.2 M) (Fig. 3e–g), as indicated by the AFM images (Fig. 3c). To support the validity of our approach in the Supplementary Information we show that the CF-DFT predictions at high concentration are very robust with respect to a significant reduction of the cation coverage of the mica at low salt concentration (Supplementary Fig. 3). This indicates that the phenomena reported here do not depend on the details of the interactions that explain the high cation coverage as shown in Fig. 3c^32^,^33^. The oscillations of the water density from the model start with a first layer of water molecules tightly bound to the adsorbed cations and the mica. In the 3D AFM images this first hydration layer cannot be distinguished from the atomic corrugation coming from mica and K atoms. For that reason it is not marked in Fig. 3a.The following layers, with a separation of about 0.3 nm in the CF-DFT model, coincide with the local maxima in 3D AFM force and identify with the oscillations observed on the experimental force curve as an effect of the second and third hydration layers. The anions are effectively excluded from the interface.

The experimental *xy* frames (Fig. 3c,d) reveal the atomic arrangement within the layered structure hinted in the *xz* frame of the 3D AFM image (Fig. 3a). Figure 3c, at *z*=0 nm, shows the hexagonal structure and atomic corrugation of the K^+^ ions (yellow dots, separated 0.52 nm) adsorbed on the cleaved mica (001) face. The K^+^ ions from the solution occupy the positions in the cleaved plane previously occupied by K^+^ ions in the bulk crystal; the water molecules from the first hydration layer are tightly bound to the solid surface, occupying the space between the cations. The second hydration layer (Fig. 3d) is placed 0.25 nm above the adsorbed K^+^ cations and it follows its atomic corrugation (0.03 nm). Our CF-DFT results agree with this interpretation; the dark hat above each cation represents the local absence of water in the first hydration layer, and the density map (Fig. 3f) gives the clear interpretation that the water molecules in the 2^nd^ layer are centred on top of the cations of the first layer, but with a larger lateral spreading.

**Figure 4:**
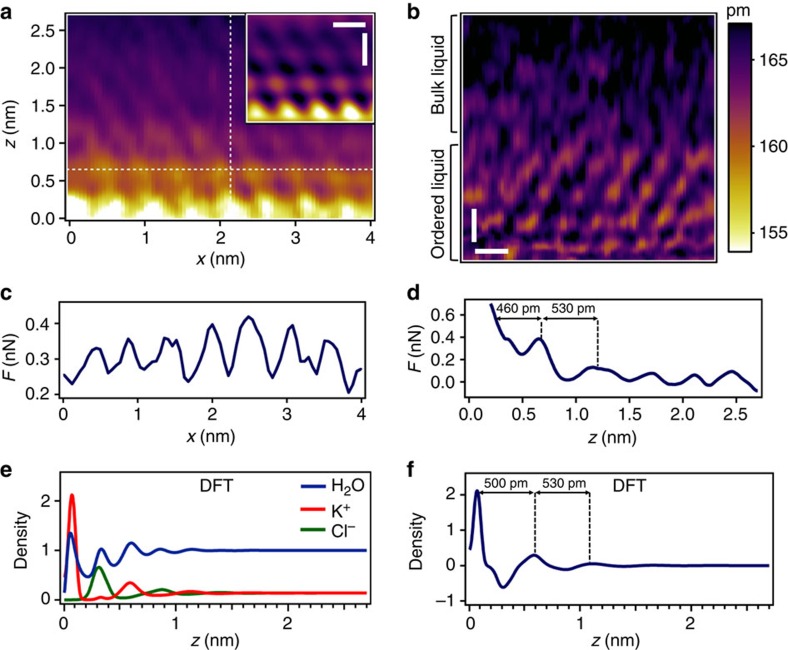
Atomic scale structure of mica-KCl (aq.) interfaces at high molarities. (**a**) *xz* frame (low pass filtered image). At high molarities the interface is characterized by the presence of an ordered liquid layer 2–3 nanometres thick. The inset shows a filtered image (FFT) of the bottom right corner of the *xz* frame. The ordered layers show atomic scale features along *x* and *z* axis. The *xz* frame shows the variations of the phase shift. (**b**) *xz* frame extending 5 nm above the mica surface (not shown). The side bar shows the variations of the oscillation amplitude during the *z* motion (*A*_0_=169 pm). (**c**) The force curve taken along the horizontal dashed line in **a** show a mean periodicity of 0.5 nm. It follows the lattice structure of the mica(001). (**d**) Force curve along the perpendicular line plotted in **a**. The force oscillates with the distance from the mica. (**e**) CF-DFT simulations at high molarities. Number density profiles perpendicular to the mica surface for K^+^, Cl^−^ and water. The density profiles are normalized with respect to the bulk number density of water. (**f**) Net charge density profile (cation minus anion). Scale bars, 0.5 nm (**a**,**b**).

At higher salt concentrations (3–5 M), the interfacial layer has a different structure and composition. It is thicker (∼3–4 nm) and shows a crystal-like structure. Figure 4a,b show some *xz* frames taken from 3D AFM images. The observed structure resembles an epitaxial growth because the mean periodicity in the *xy* plane is 0.51 nm (Fig. 4c), which is very close to the mica(001) lattice parameter (0.52 nm). In the perpendicular direction (Fig. 4d), the force curve shows a decaying oscillatory behaviour. The atomic size features observed in the *xz* frames indicate the discrete character of the interface in all the spatial coordinates. Remarkably atomic size features are still observed 2 nm above the mica surface.

The CF-DFT results near a saturated salt concentration (about 5 M in our CF-DFT model) show that the number density profiles for K^+^ and Cl^−^ have strong oscillations as function of the distance from the mica surface (Fig. 4e). Three peaks are clearly observed for each ion, and over that region water molecules are only partially depleted. The contrast mechanism observed in the 3D AFM images at high concentrations is still unknown. The separation among the local maxima in the experimental tip-liquid force (Fig. 4d) is in good agreement with the separation among the local maxima in the net charge profile (Fig. 4f). This observation together with decaying character of the force as a function of the distance from the mica surface suggests a correlation with the global cation–anion 3D structure, rather than with the plain total number density of liquid molecules. The 3D structure in the liquid solution propagates from the mica surface and it cannot be understood without a cooperative effect between the three chemical species. It involves the arrangement of alternating cation and anion planes parallel to the mica surface, still with a significant amount of water, being clearly different from the KCl crystal that may be obtained in the same CF-DFT model at coexistence with a saturated water solution. As presented in Supplementary Information, the epitaxial crystal found in our CF-DFT model contains very little water inside, as expected in a real KCl crystal. Thick layers of that epitaxial crystal covering the mica can also be obtained in our CF-DFT calculations, but they imply a strong reorganization of the first density layers, and correspond to separated minima of the grand potential energy.

Experimentally, we also observe the formation of small nanocrystals on the mica at saturation. Supplementary Fig. 4 shows atomic resolution images of NaCl and KCl crystals deposited from the solution. They were obtained in the presence of a saturated electrolyte concentration. The complete evaporation of the water from the fluid cell left the mica covered by salt nano or microcrystals (Supplementary Fig. 5). The morphology of the nano and microcrystals follows some of the crystalline orientations of the mica^40^.

## Discussion

Our findings establish three different structures for the interfacial layer formed by alkali halide electrolyte solutions interacting with a mica surface. In pure water and at very low molarities (≤10 mM) is very difficult to image the hydration layers. This is due to the diffusive force associated with the negative charge of the mica surface. At low molarities, a monolayer of alakali cations is adsorbed on the mica. A few water monolayers are layered above them. At high molarities (3–5 M), an ordered liquid layer several nanometres thick is formed. The lateral periodicity follows the mica lattice parameter while the vertical periodicities are controlled by the interactions between the ions and the water molecules. At saturation, ionic crystals precipitate on the surface of mica.

A central issue of these observations concerns the physical state of the ordered layer, crystalline or liquid. To capture the 3D AFM images, the tip moves up and down into the mica–liquid interface with a frequency of 100 Hz. In the process the tip continuously disrupts and pushes away the ions and water molecules of the ordered layer. This process supports a liquid-like character for the ordered layer. We explain the compatibility of a crystal-like structure with a liquid-like deformability by noting the different time scales involved in the 3D imaging process and the ion and water molecule motions. A single event in the tip motion happens in the microsecond range, this is, several orders of magnitude larger than the expected times for the motion of individual water molecules and ions (∼10^−10^ s). Thus the 3D AFM images provide the average positions of the ions and water molecules at the millisecond to second time scales.

The role of the tip geometry and composition in atomic resolution experiments in liquid is being studied by molecular dynamics simulations^41^. Ions and hydration layers should also be formed on the tip. However, the ordering of the tip's interfacial layer should be weaker than the one found on mica because the tip's nanoscale roughness. We propose that the 3D AFM images represent the structure of the ordering of electrolytes on the mica with little distortion from the tip. This interpretation is consistent with the agreement observed between the experiments and the theory, which represents the equilibrium 3D density distributions in the unperturbed mica-liquid interface.

Force microscopy has provided atomic and molecular resolution images of a large variety of surfaces from crystalline systems^42^ to biomolecules^43^. The combination of atomic resolution with a genuine *xyz* probe motion opens the way to investigate the 3D atomic structure and interactions of ions and solvent molecules near solid surfaces with a few nanometres depth. These findings could serve as the model for the structure of electrolyte interfacial layers in the vicinity of hydrophilic surfaces with relevance in a large variety of fields from surface electrochemistry to molecular and cell biology.

## Methods

### 3D AFM

The 3D AFM images of solid-liquid interfaces were obtained by introducing a sequential 3D motion for the tip. This motion is independent of the tip oscillation. The 3D tip motion involves a sinusoidal signal added to piezo *z*, and a modification of the *xy* tip motion to acquire data points on either of trace or retrace but not in both. The 3D AFM was operated in amplitude modulation AFM^35^ by using a ‘loose' feedback control. This has been achieved by decreasing the bandwidth of the *z* feedback loop between 1–2 kHz. This enables tip-sample plane tilt correction without affecting the sinusoidal movement. The free oscillation amplitudes *A*_0_ were in the 35–190 pm range and the set-point amplitude was set at a 0.9–0.95 of *A*_0_. The oscillation of the cantilever was driven by photothermal excitation at a wavelength of 405 nm. The 3D AFM was implemented in a commercial AFM platform (Cypher, Asylum Research, Oxford Instruments).

### 3D motion

The AFM images involve a maximum frequency of 50 kHz per data point. For images with a *z* depth equal or below 1 nm (Fig. 2a), the *z* displacement is performed at a frequency of 100 Hz with a sampling performed in both directions, approach and retract from the sample (512 data points in total); the *x* displacement is performed with a frequency of 1.22 Hz and the sampling involves 80 data points; the *y* displacement is performed with a frequency of 0.02 Hz and the sampling involves 64 data points. For 3D images with a *z* depth above 2 nm (Fig. 2b,c), the *z* displacement is performed at a frequency of 50 Hz with a sampling of 1,024 data points (512 approach and 512 retract); the *x* displacement is performed at a frequency of 0.61 Hz with a sampling of 80 data points and the *y* displacement is performed at a frequency of 0.01 Hz with a sampling of 64 data points.

### Microcantilevers

We have used silicon cantilevers PPP_NCHAu_D (Nanosensor, Germany). The force constant, resonant frequency and quality factors of the first two eigenmodes were determined in liquid. Their values were in the following range, *k*_1_=22–40 N m^−1^ *f*_1_=120–180 KHz, *k*_2_= 800–1,600 N m^−1^ *f*_2_=800–1,100 KHz, *Q*_1_=7–12, *Q*_2_=18–25. The above values have been determined in water. The force constants have been determined by using the thermal method.

### Sample preparation

Discs of muscovite mica (SPI supplies, USA) were cleaved with adhesive tape before each experiment. For aqueous NaCl solution experiments, the muscovite mica was thoroughly rinsed with pure water (18.2 MΩ) before introducing it in the AFM chamber. NaCl and KCl solutions were prepared from 99.5% (NaCl) and 99.0% (KCl) salts (Sigma-Aldrich) dissolved in ultrapure water (18.2 MΩ, pH=5.5).

### Classical Fluid Density Functional Theory

We use the DF formalism, which is the most successful theoretical framework to get a consistent description of the molecular structure and the thermodynamics of interfacial liquid systems^14^,^44^. We model the system as a ternary liquid mixture, with a solvent (water) as major component (bulk density *ρ*_*s*_), and equal concentrations of two solute species (cations and anions), with equal bulk densities, *ρ*_*c*_=*ρ*_*a*_, given by the fraction *X*=*ρ*_*c,a*_*/ρ*_*t*_ over the total density. The molecular packing effects are obviously at the core of the 3D molecular structure, and they are included in our model as Hard Sphere impenetrability of the molecules. We have taken different Hard Sphere diameters for the three species; for the ions twice its ionic radius^45^, *σ*_Cl_=0.362 and *σ*_K_=0.266 and *σ*≡*σ*_Water_=0.2569, nm for the water. In a previous simplified model we had used the same molecular diameter for all the species, *σ*, that fixes the length scale in the model, and the good qualitative comparison between the two models provides an important clue. The size differences between the ions, as well as the anisotropy of water, would certainly be important for a quantitative prediction, but as shown by our results, these effects are not needed to understand the qualitative aspects of the experimental observations. What is crucial is to have a good DF description of the molecular packing for the extremely inhomogeneous density distributions of the first cation layer on the mica. Therefore, we use the dimensional interpolation fundamental measure theory-free energy, which reproduces very accurately the crossover from 0D cavities, to 3D bulk liquids and to the crystallization of the Hard Sphere system^46^,^47^. Additional description is provided in Supplementary Figs 6–7 and Supplementary Methods.

### Data availability

The data that support the findings of this study are available from the corresponding author on request.

## Additional information

**How to cite this article:** Martin-Jimenez, D. *et al*. Atomically resolved three-dimensional structures of electrolyte aqueous solutions near a solid surface. *Nat. Commun.* 7:12164 doi: 10.1038/ncomms12164 (2016).

## Supplementary Material

Supplementary Information Supplementary Figures 1-7, Supplementary Methods and Supplementary References.

Supplementary Movie 1 3D AFM image of the mical-KCl aqueous solution (0.2 M) was obtained in amplitude modulation AFM by exciting the cantilever at 809 kHz (2nd eigenmode); *k*= 855 N/m, *Q*= 20; *A*_0_= 0.04 nm. Data points= 1310720.

Supplementary Movie 2 3D AFM image of the mical-KCl aqueous solution (~ 4 M) was obtained by exciting the cantilever at 838 kHz (2nd eigenmode); *k*= 921 N/m, *Q*= 24; *A*_0_= 0.11 nm. Data points= 1984000.

Supplementary Movie 3 3D AFM image of the mical-NaCl aqueous solution (~ 5 M) was obtained by exciting the cantilever at 159 kHz (1st eigenmode), *k*= 24 N/m, *Q*= 7; *A*_0_= 0.23 nm. Data points = 2400000.
