# Supplementary material for: Atomically resolved three-dimensional structures of electrolyte aqueous solutions near a solid surface
Source: Nat Commun. 2016 Jul 15;7:12164. doi: 10.1038/ncomms12164 (PMC4947176; doi:10.1038/ncomms12164)
Supplement: Supplementary Information — Supplementary Figures 1-7, Supplementary Methods and Supplementary References. [file ncomms12164-s1.pdf]

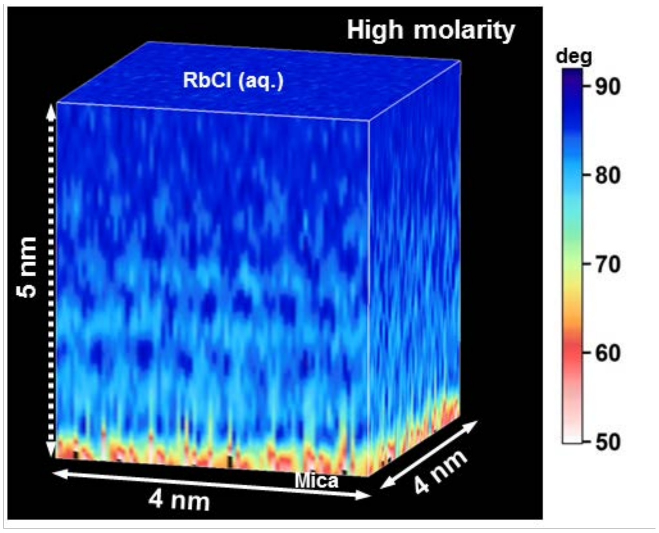

Supplementary Figure 1. **Three-dimensional atomic resolution image of mica-RbCl solution interfaces.**. 3D AFM image of a mica-RbCl (aq.) interface (~6 M). The interface is divided in two main regions, an ordered liquid layer extending up to 3 from the mica surface and the bulk solution above it. The 3D AFM image shows the variations of the phase shift as a function of the tip's position (*x*,*y*,*x*).

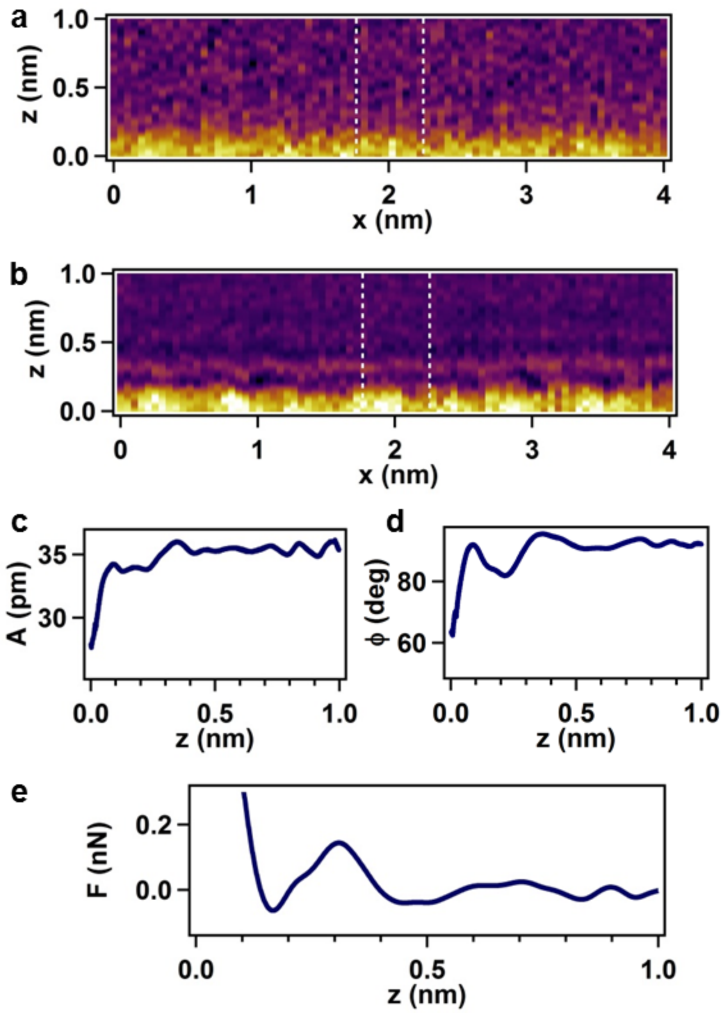

Supplementary Figure 2. **Main steps to reconstruct the force from amplitude modulation AFM data**. **a,** *xz* map showing variations in the oscillation amplitude. **b,** *xz* map showing variations in the phase shift. **c,** Amplitude dependence on the tip-mica separation (amplitude curve). The values are averaged over the region marked by dashed lines in **a**. **d,** Phase shift dependence on the tip-mica separation (phase shift curve). The values are averaged over the region marked by dashed lines in **b**. **e,** The amplitude and phase shift curves are the inputs required to reconstruct the force curve[1,2].

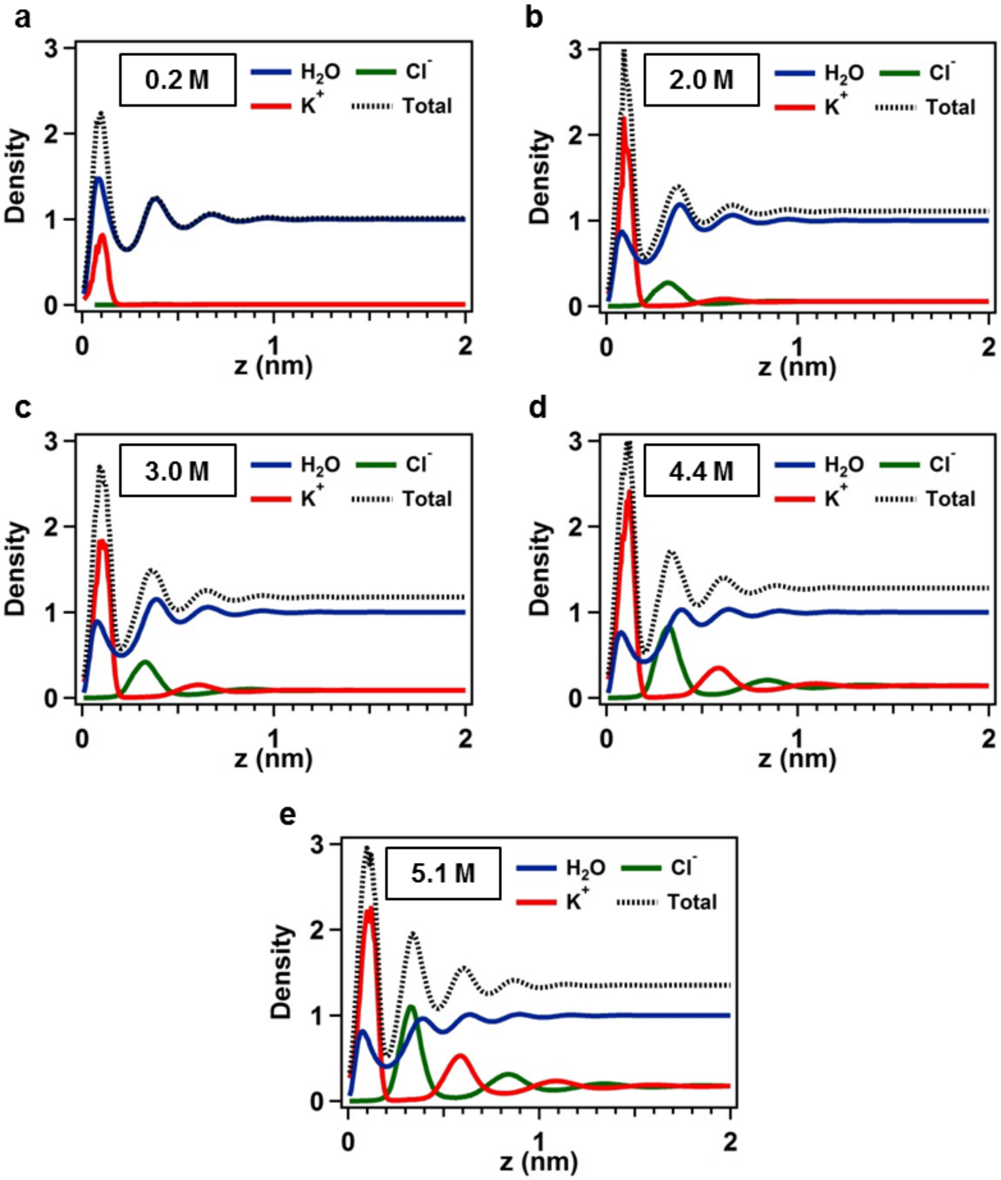

Supplementary Figure 3. CF-DFT profiles for different molarities. a-e, CF-DFT results of the number density perpendicular to the mica surface (*z* direction), for several salt concentrations. In this case the same HS radii has been used for the three species (water, cations and anions). At each *z* point, the density is averaged over the corresponding *xy* plane. The density profiles are normalized with respect to the bulk number density of water. In these figures, a 50% of the coverage of the mica by Potassium at low salt concentration (0.2 M) has been considered. This is in contrast to the 90% coverage shown in Fig. 3e. However, the trend observed at high molarities (4.4 M) is independent of the initial cation coverage and of the approximation used for the HS radii.

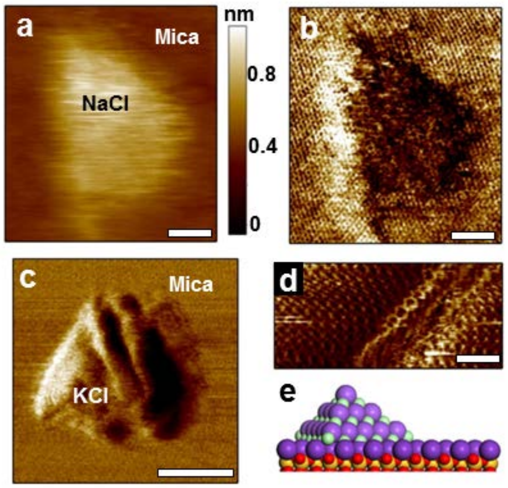

Supplementary Figure 4. **Nanoscale alkali halide crystals on mica**. At saturation values, we observe formation of small crystals on the mica. **a-b,** AFM images of NaCl crystal on mica (Topography and phase shift images, respectively). **c,** AFM top view of a small KCl structure on mica. **d,** A high resolution phase image reveals the atomic lattice of the KCl (111). Several atomically-flat terraces are imaged. **e,** Scheme of the KCl crystal structure on muscovite mica. Purple (K), light green (Cl), red (O) and yellow (Si or Al 3:1). Scale bars, 5 nm (**a**, **b**), 50 nm (**c**), 2 nm (**d**).

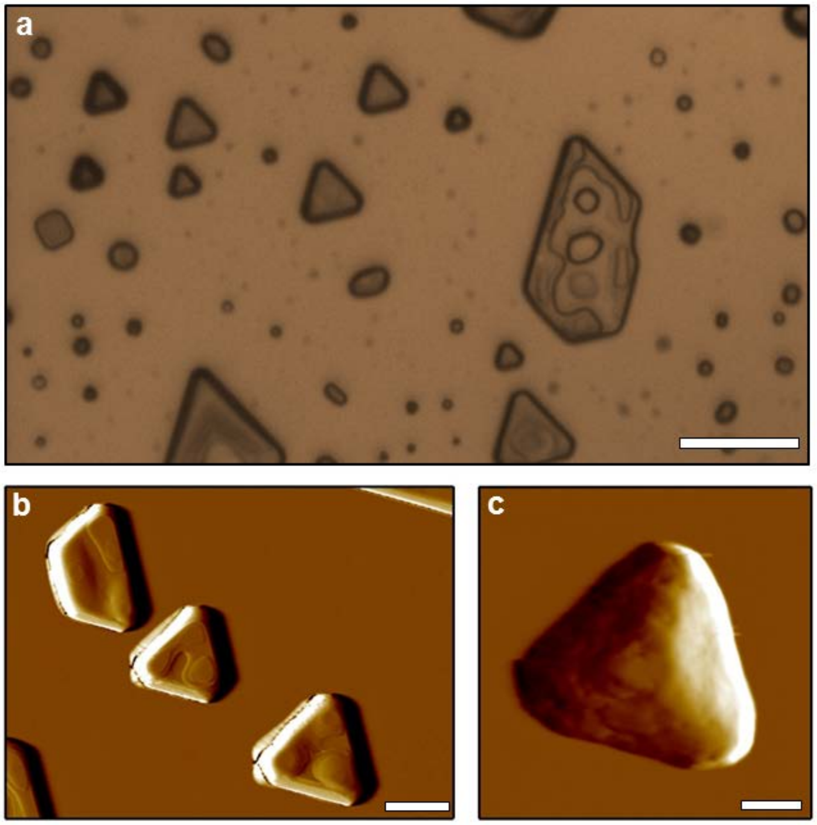

Supplementary Figure 5. KCl crystals structures on mica after the full evaporation of water. a, Optical image of micrometer and submicrometer ionic crystals on mica, The crystals show regular shapes that follow the preferred crystallographic orientations of the mica. **b-c,** AFM images (deflection channel) of KCl crystals on mica. The above AFM images were acquired in air and contact mode with a cantilever RC-800PSA (Olympus), Nominal k= 0.76 N/m. Scale bars, 10 µm (**a**), 2 µm (**b**) and 200 nm (**c**).

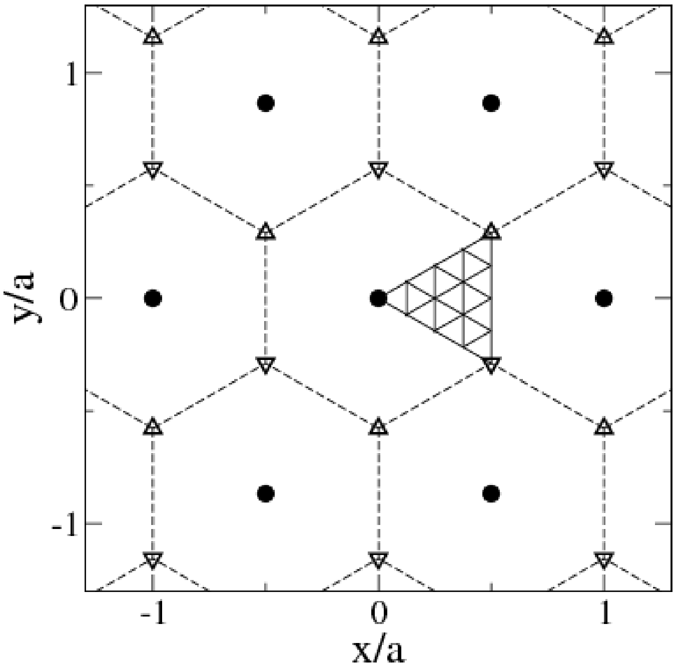

Supplementary Figure 6. Scheme of the numerical discretization used to minimize the grand-potential energy as functional of density distributions. The cation adsorbing sites (full circles) form a triangular lattice on the *xy* plane with nearest neighbor distance $\boldsymbol{a}$. The dashed lines are the boundaries of the unit cells in that plane. The density distributions $\boldsymbol{\rho_\alpha(x, y, z)}$, with $\boldsymbol{\alpha = w, c, a}$ for water, cations and anions respectively, are described as linear functions of *x*, *y* and *z* within the prisms with triangular base on the *xy* plane given by the mesh of full lines, with separation $\Delta\boldsymbol{x} = \frac{\boldsymbol{a}\sqrt{\boldsymbol{3}}}{\boldsymbol{4}}$ and with a height $\Delta\boldsymbol{z} = \frac{\boldsymbol{a}\sqrt{\boldsymbol{3}}}{\boldsymbol{40}}$ on the *z* axis. We have used a finer grid for some cases, with no relevant changes in the density profiles.

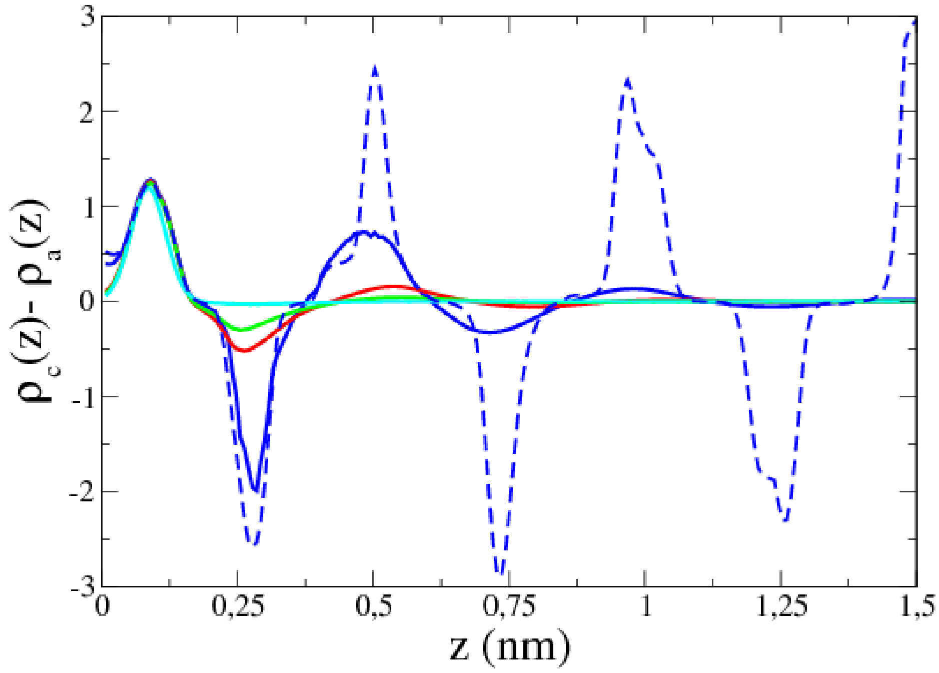

Supplementary Figure 7. Cation density profiles minus the anion density profiles for $a/\sigma=1.05$ and $u/k_BT=250$ at several salt concentrations for the model with the same HS radii for the three species (water, cations and anions). The full lines show the "direct" liquid interface regime; Cyan: 0.27M, green: 2.7M, red: 5.5M, and blue: 6.1M. The blue dashed line is the metastable epitaxial crystal layer for the higher salt concentration.

Supplementary methods

Our theoretical Classical Fluid Density Functional (CF-DF) calculation is useful to explore the generic aspects in the molecular structure of the liquid solution near the substrate, which is observed in the 3D-AFM maps. We do not aim to a full quantitative description of the experimental results, nor to the most realistic description of the ionic solutions, but rather to reproduce the phenomena qualitatively, with the simplest possible model, as a test of its generality, and for a better understanding of its origin.

The ionic interactions are qualitatively important to set the equilibrium crystal structure, and it is included with a mean-field approximation[3,4] for an effective Yukawa pair potential $\varphi(r) = \pm u\, e^{-\kappa r}/(4\pi\kappa r)$. This interaction potential acts beyond the HS core ($r \geq \sigma$), with repulsion (+ sign) between equal ions and attraction (− sign) between unequal ion species. All the results presented here correspond to the choice $\sigma\kappa=4$, which gives a reasonable ratio of 3.7 between the attraction from the six nearest neighbors (at distance σ) repulsion from the twelfth next-nearest neighbors. The parameter u sets the strength of the ionic interactions, relative to the (room temperature) thermal energy $k_B T$. At high salt concentration, $X \gtrsim 0.1$ (the precise value depending on u), a salt crystal forms in the liquid solution, as the global minimum of the CF-DF grand potential $\Omega[\rho_w(\vec{r}), \rho_c(\vec{r}), \rho_a(\vec{r})]$. This is in fair agreement with the room temperature solubility of KCl and NaCl (between 4 and 6 M), which correspond to molar fractions $X \approx 0.08 - 0.12$.

The mica surface is represented by an impenetrable wall for all the species for $z \leq 0$, while for $z > 0$ it acts as a smooth repulsive potential on water and the anions, $V_{w,a}(\vec{r}) = V_{w,a}^{(o)} \exp(-\tau_{w,a}\, z)$, with $V_w^{(o)} = 6\, k_B T$ and $\tau_w\, \sigma = 8$ for water. The repulsion is stronger, $V_a^{(o)} = 12\, k_B T$, and longer ranged, $\tau_a \sigma = 2$, for the anions, to take into account the net negative charge of the mica surface. The specific adsorption sites for the cations are modelled by

a 3D external potential $V_c^{(o)}(\vec{r})$ built to produce narrow Gaussian peaks, with nearly complete (0.95 for $X = 0.05$) mean occupancy at the sites of a triangular lattice with nearest neighbor distance $2a$ as shown in Supplementary Fig. 6. The numerical minimization of the CF-DF grand-potential energy takes advantage of the mica symmetry, but still allowing for a possible symmetry breaking of an epitaxial crystal growing with the (111) face on the mica plane. At low molar fraction, $X = 0.05$, the interfacial structure is limited to a strongly structured absorbed monolayer of cations and water, followed by a few hydration layers (Fig. 3e). We use the results of the realistic model simulation in this regime[5] to tune the parameters of wall potential and the effective bulk water density, the later set to $\rho_w\sigma^3 = 0.6$ to get a similar decay of the water layers.

The minimization of $\Omega[\rho_w(\vec{r}), \rho_c(\vec{r}), \rho_a(\vec{r})]$ (see the sketch in Supplementary Fig. 6) gives qualitatively similar results for different ionic strengths (over the range $225<u/k_BT<350$ explored here), and ratios between the semi-distance of the cation adsorbing sites on the mica and the HS diameter in the model (over the range $1.05 \leq a/\sigma \leq 1.20$). The growth of a thick epitaxial crystalline structure is always observed for large salt concentrations (Fig. 4f), while for very low concentrations the density profiles have an adsorbed cation monolayer followed by a few water layers, weakly structured. These two structures correspond to two separated minima of $\Omega[\rho_w(\vec{r}), \rho_c(\vec{r}), \rho_a(\vec{r})]$, this is to a first order wetting transition between the "direct" mica-liquid interface and the epitaxial crystal layer. The strength of this first order transition depends on the parameter choice. All the relevant results presented in the main text, for the comparison with the 3D-AFM images, are within the "direct" liquid interface regime. This is the regime observed in our CF-DF results over a broad range of salt concentrations (Supplementary Fig. 7), smoothly developing a deeper 3D structured region of cations and anions as the salt

concentration increases. In our CF-DF model, these structured-fluid surfaces are thermodynamically separated from the epitaxial crystal (depicted also in Supplementary Fig. 7), and they persist as a meta-stable local minima of $\Omega[\rho_w(\vec{r}), \rho_c(\vec{r}), \rho_a(\vec{r})]$ even for salt concentrations that make the epitaxial crystal layer to be stable state of the surface.

The calculations shown in the main text the interaction with the mica is modelled to have a nearly complete monolayer of cations at low salt concentrations (0.2 M) (Fig. 3e-g), as indicated by the AFM images (Fig. 3c). The Supplementary Fig. 3 shows that the DFT predictions at high concentration are very robust with respect to a significant reduction of the cation coverage of the mica at low salt concentration. Those results emphasize that the observed phenomena does not require to break charge neutrality at the mica-cation interface. Besides the results of Supplementary Fig. 3 have been obtained using our simplified model with the same molecular diameter for all the species. The similarity of the results shows that the size differences between the ions, or other molecular details like the anisotropy of water, would certainly be important for a quantitative prediction, but these effects are not needed to understand the qualitative aspects of the experimental observations.

Supplementary References

1. Holscher, H. Quantitative Measurement of Tip-Sample Interactions in Amplitude Modulation Atomic Force Microscopy. *Appl. Phys. Lett.* **89,** 3 (2006).
2. Payam, A. F., Martin-Jimenez, D., & Garcia, R. Force reconstruction from tapping mode force microscopy experiments. *Nanotechnology* **26**, 185706 (2015).
3. Lowen, H. Density functional theory of inhomogeneous classical fluids: recent developments and new perspectives. *J. Phys. Cond. Matter* **46** , 11897-11905 (2002)
4. Evans, R. The nature of the liquid-vapour interface and other topics in the statistical mechanics of non-uniform, classical fluids. *Adv. Phys.* **28**, 143 (1979).
5. Siretanu, I. *et al.* Direct observation of ionic structure at solid-liquid interfaces: a deep look into the Stern Layer. *Scientific Rep.* **4**, 4956 (2014).
